# The genome sequence of the banded centipede, *Lithobius variegatus *Leach, 1814

**DOI:** 10.12688/wellcomeopenres.24329.1

**Published:** 2025-06-02

**Authors:** Gregory D. Edgecombe, Liam M. Crowley, Mark G. Telfer, Lauren Hughes, University of Oxford and Wytham Woods Genome Acquisition Lab, Natural History Museum Genome Acquisition Lab, Darwin Tree of Life Barcoding collective, Wellcome Sanger Institute Tree of Life Management, Samples and Laboratory team, Wellcome Sanger Institute Scientific Operations: Sequencing Operations, Wellcome Sanger Institute Tree of Life Core Informatics team, Tree of Life Core Informatics collective, Darwin Tree of Life Consortium

**Affiliations:** 1 Natural History Museum, London, England, UK; 2 University of Oxford, Oxford, England, UK; 3 Independent researcher, Ventnor, Isle of Wight, England, UK

**Keywords:** Lithobius variegatus, centipede, genome sequence, chromosomal, Lithobiomorpha

## Abstract

We present a genome assembly from a female specimen of *Lithobius variegatus* (banded centipede; Arthropoda; Chilopoda; Lithobiomorpha; Lithobiidae). The assembly contains two haplotypes with total lengths of 1,766.49 megabases and 1,768.00 megabases. Most of haplotype 1 (97.68%) is scaffolded into 23 chromosomal pseudomolecules. Haplotype 2 was assembled to scaffold level. The mitochondrial genome has also been assembled, with a length of 17.44 kilobases.

## Species taxonomy

Eukaryota; Opisthokonta; Metazoa; Eumetazoa; Bilateria; Protostomia; Ecdysozoa; Panarthropoda; Arthropoda; Mandibulata; Myriapoda; Chilopoda; Pleurostigmophora; Lithobiomorpha; Lithobiidae; *Lithobius*; *Lithobius variegatus* Leach, 1814 (NCBI:txid60158)

## Background

*Lithobius variegatus* ( Leach, 1813) was long thought to be endemic to Britain and Ireland but was eventually discovered in the Channel Islands, Brittany and the northwest Iberian Peninsula ( Eason & Serra, 1986). In France it is now known from scattered parts of the North-West/Armorica and Pyrénées Atlantique in the southwest ( Iorio, 2014). In both Britain and Ireland, *Lithobius variegatus* is widely distributed but is missing in much of eastern England and Scotland, where it has a marked western tendency.

It is a predominantly rural species, with relatively few urban records. Deciduous woodland is its principal habitat, although it is also commonly found in mixed woodland, grassland, and heath or moors ( Barber, 2022). It is collected less frequently in maritime sites, parks and gardens, waste ground, and wetlands. It had a broad altitudinal range, mostly at less than 250 m but found at up to 987 m. Its most typical microsites, in descending frequency, are under stones, under dead wood, under their bark, and in leaf litter. Collected year-round, April and May provide the most records, though *L. variegatus* is common through September ( Barber, 2022). Egg-laying peaks in early summer, maturity is reached the next summer, and the duration of life is approximately two years ( Roberts, 1956). Larval and post-larval stadia were described and figured by Eason (1964). Studies of gut content from English woodland show that immature stadia of *L. variegatus* feed predominantly on Collembola whereas adults take (in descending order) collembolans, spiders, mites, opilions, and molluscs ( Roberts, 1956). It is known to be arboreal ( Eason, 1964).

In life, *Lithobius variegatus* is easily identified by its colour, the pale brown tergites being marbled with dark violet and the legs having alternating pale and dark bands ( Barber, 2008; Barber, 2009). Its distinctive habit of remaining motionless when disturbed may be related to its colouration providing camouflage ( Eason, 1964). British specimens reach body length of 24 mm, Irish ones being up to 30 mm. Other distinguishing characters are numerous (6+6 to 7+7) teeth on a very broad, prominent anterior border of the forcipular coxosternite and the presence of small projections on tergite 7 (unique among British species) along with projections on tergites 9, 11 and 13.

Few whole genome sequences for centipedes have been generated, these including *Strigamia maritima* (GCA_000239455.1) ( Chipman *et al.*, 2014) and *S. acuminata* (GCA_949358305.1) ( Edgecombe *et al.*, 2023), *Scolopendra mutilans* ( Zhang *et al.*, 2024), and *Lithobius niger* (GCA_023313725.1), *Rhysida immarginata* (GCA_023313115.1), and *Thereuonema tuberculata* (GCA_023159025.1) ( So *et al.*, 2022). The MetaInvert database at Senckenberg Görlitz and the LOEWE Centre for Translational Biodiversity Genomics also offers genome sequences for 19 Chilopoda, among them seven species of *Lithobius* ( Collins *et al.*, 2023).

**Figure 1.**
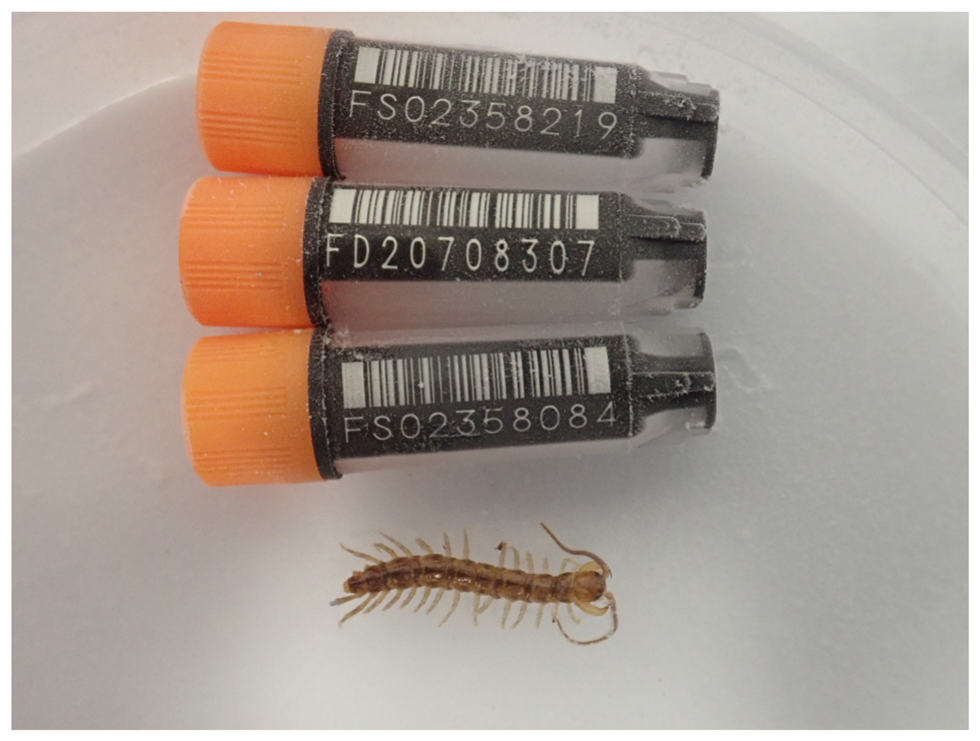
Photograph of the *Lithobius variegatus* (qcLitVari1) specimen used for genome sequencing.

The genome of *Lithobius variegatus* ( Figure 1) was sequenced as part of the Darwin Tree of Life Project, a collaborative effort to sequence all named eukaryotic species in the Atlantic Archipelago of Britain and Ireland. Here we present a chromosomally complete genome sequence for *Lithobius variegatus*, based on one female specimen from Wytham Woods, England, UK (latitude 51.4680, longitude –0.2338).

## Genome sequence report

### Sequencing data

The genome of a specimen of *Lithobius variegatus* ( Figure 1) was sequenced using Pacific Biosciences single-molecule HiFi long reads, generating 101.84 Gb (gigabases) from 11.72 million reads. GenomeScope analysis of the PacBio HiFi data estimated the haploid genome size at 1,575.48 Mb, with a heterozygosity of 1.31% and repeat content of 36.49%. Based on this estimated genome size, the sequencing data provided approximately 60 coverage of the genome. Chromosome conformation Hi-C sequencing produced 152.69 Gb from 1,011.17 million reads. Table 1 summarises the specimen and sequencing information.

**Table 1.** Specimen and sequencing data for *Lithobius variegatus*.

Project information			
**Study title**	Lithobius variegatus (banded centipede)		
**Umbrella BioProject**	PRJEB77497		
**Species**	*Lithobius variegatus*		
**BioSpecimen**	SAMEA8603223		
**NCBI taxonomy ID**	60158		
Specimen information			
**Technology**	**ToLID**	**BioSample accession**	**Organism part**
**PacBio long read sequencing**	qcLitVari1	SAMEA8603811	anterior_body
**Hi-C sequencing**	qcLitVari1	SAMEA8603809	head
**RNA sequencing**	qcLitVari2	SAMEA9066090	mid_body
Sequencing information			
**Platform**	**Run accession**	**Read count**	**Base count (Gb)**
**Hi-C Illumina NovaSeq 6000**	ERR13363434	1.01e+09	152.69
**PacBio Revio**	ERR13362651	7.25e+06	59.94
**PacBio Sequel IIe**	ERR13362653	1.33e+06	12.29
**PacBio Sequel IIe**	ERR13362654	2.60e+06	21.0
**PacBio Sequel IIe**	ERR13362652	5.37e+05	8.61
**RNA Illumina HiSeq 4000**	ERR13363433	3.03e+07	4.58

### Assembly statistics

The genome was assembled into two haplotypes using Hi-C phasing. Haplotype 1 was curated to chromosome level, while haplotype 2 was assembled to scaffold level. The assembly was improved by manual curation, which corrected 166 misjoins or missing joins. These interventions reduced the total assembly length by 0.82%, decreased the scaffold count by 5.88%, and increased the scaffold N50 by 4.7%. The final assembly has a total length of 1,766.49 Mb in 623 scaffolds, with 1,092 gaps, and a scaffold N50 of 78.18 Mb ( Table 2).

**Table 2.** Genome assembly data for *Lithobius variegatus*.

Genome assembly	Haplotype 1	Haplotype 2
Assembly name	qcLitVari1.hap1.1	qcLitVari1.hap2.1
Assembly accession	GCA_965125955.1	GCA_965122155.1
Assembly level	chromosome	scaffold
Span (Mb)	1,766.49	1,768.00
Number of contigs	1,715	2,106
Number of scaffolds	623	1,056
Longest scaffold (Mb)	271.1	-
Assembly metrics (benchmark)	Haplotype 1	Haplotype 2
Contig N50 length (≥ 1 Mb)	2.69 Mb	2.65 Mb
Scaffold N50 length (= chromosome N50)	78.18 Mb	75.84 Mb
Consensus quality (QV) (≥ 40)	62.4	62.2
*k*-mer completeness	77.94%	77.82%
Combined *k*-mer completeness (≥ 95%)	99.30%	
BUSCO * (S > 90%; D < 5%)	C:97.7%[S:96.4%,D:1.3%], F:1.6%,M:0.7%,n:1013	C:97.2%[S:96.1%,D:1.2%], F:1.9%,M:0.9%,n:1013
Percentage of assembly mapped to chromosomes (≥ 90%)	97.68%	-
Sex chromosomes (localised homologous pairs)	Not identified	-
Organelles (one complete allele)	Mitochondrial genome: 17.44 kb	-

* BUSCO scores based on the arthropoda_odb10 BUSCO set using version 5.5.0. C = complete [S = single copy, D = duplicated], F = fragmented, M = missing, n = number of orthologues in comparison

**Figure 2.**
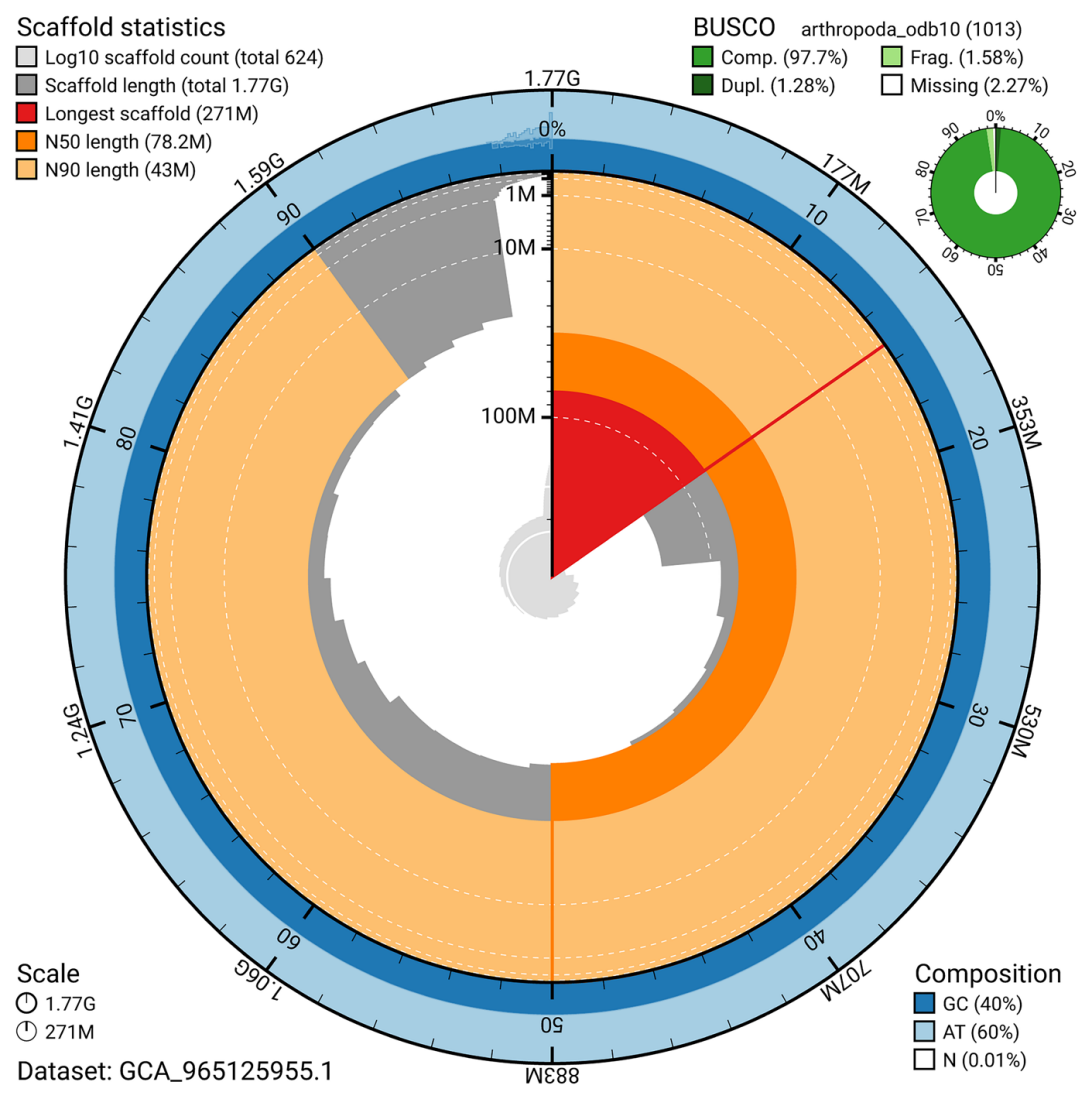
Genome assembly of *Lithobius variegatus*, qcLitVari1.hap1.1: metrics. The BlobToolKit snail plot provides an overview of assembly metrics and BUSCO gene completeness. The circumference represents the length of the whole genome sequence, and the main plot is divided into 1,000 bins around the circumference. The outermost blue tracks display the distribution of GC, AT, and N percentages across the bins. Scaffolds are arranged clockwise from longest to shortest and are depicted in dark grey. The longest scaffold is indicated by the red arc, and the deeper orange and pale orange arcs represent the N50 and N90 lengths. A light grey spiral at the centre shows the cumulative scaffold count on a logarithmic scale. A summary of complete, fragmented, duplicated, and missing BUSCO genes in the arthropoda_odb10 set is presented at the top right. An interactive version of this figure is available at https://blobtoolkit.genomehubs.org/view/GCA_965125955.1/dataset/GCA_965125955.1/snail.

**Figure 3.**
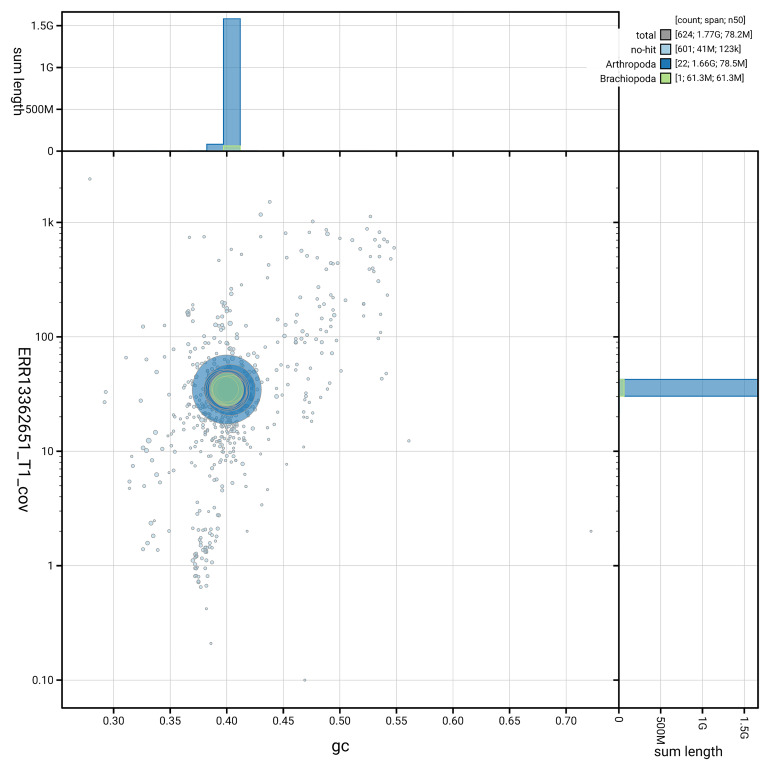
Genome assembly of *Lithobius variegatus*, qcLitVari1.hap1.1: BlobToolKit GC-coverage plot. Blob plot showing sequence coverage (vertical axis) and GC content (horizontal axis). The circles represent scaffolds, with the size proportional to scaffold length and the colour representing phylum membership. The histograms along the axes display the total length of sequences distributed across different levels of coverage and GC content. An interactive version of this figure is available at https://blobtoolkit.genomehubs.org/view/GCA_965125955.1/dataset/GCA_965125955.1/blob.

**Figure 4.**
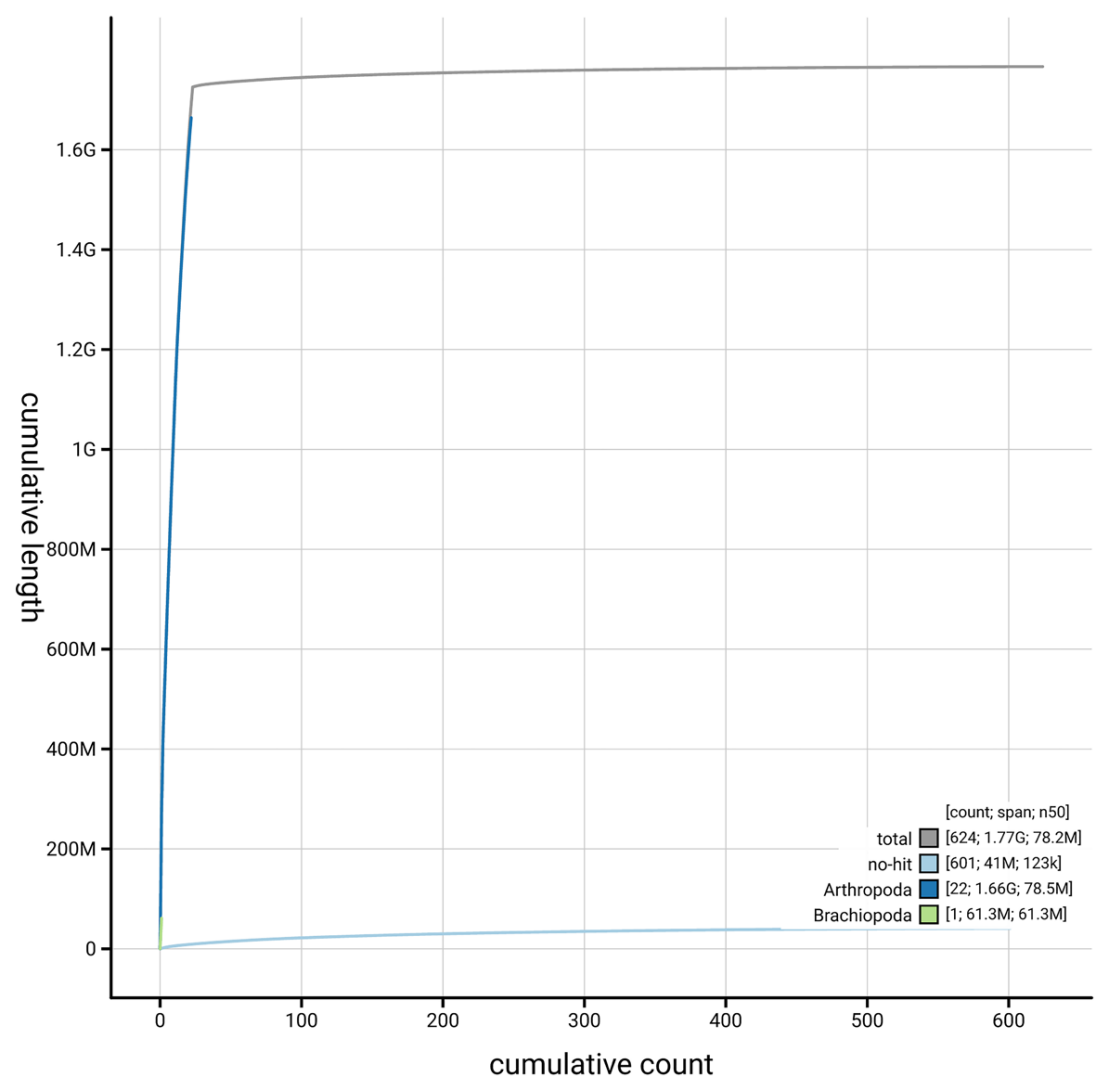
Genome assembly of *Lithobius variegatus,* qcLitVari1.hap1.1: BlobToolKit cumulative sequence plot. The grey line shows cumulative length for all scaffolds. Coloured lines show cumulative lengths of scaffolds assigned to each phylum using the buscogenes taxrule. An interactive version of this figure is available at https://blobtoolkit.genomehubs.org/view/GCA_965125955.1/dataset/GCA_965125955.1/cumulative.

The snail plot in Figure 2 provides a summary of the assembly statistics, indicating the distribution of scaffold lengths and other assembly metrics. Figure 3 shows the distribution of scaffolds by GC proportion and coverage. Figure 4 presents a cumulative assembly plot, with separate curves representing different scaffold subsets assigned to various phyla, illustrating the completeness of the assembly.

**Figure 5.**
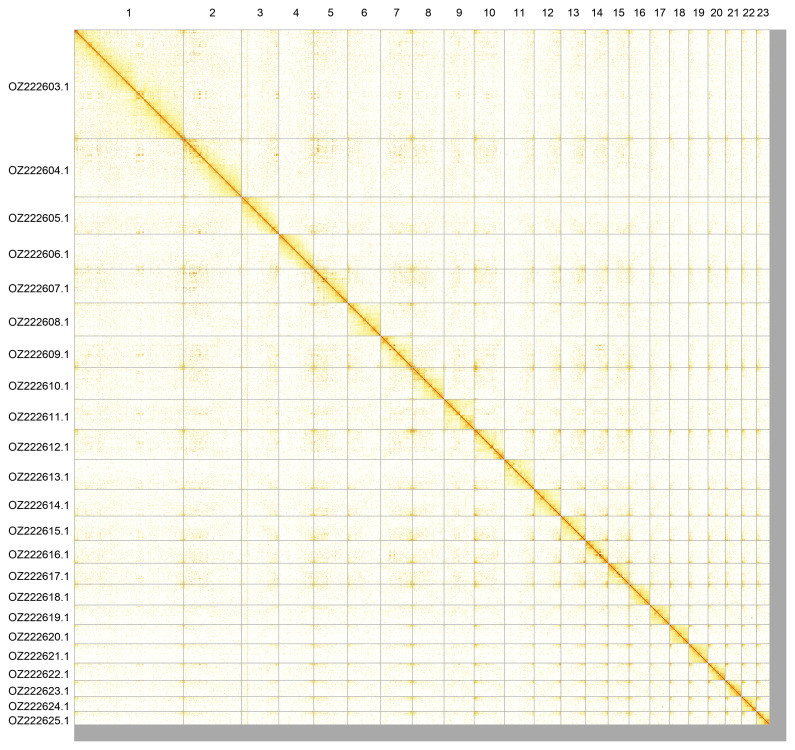
Genome assembly of *Lithobius variegatus,* qcLitVari1.hap1.1: Hi-C contact map of the qcLitVari1.hap1.1 assembly, produced in PretextView. Chromosomes are shown in order of size from left to right and top to bottom.

Most of the assembly sequence (97.68%) was assigned to 23 chromosomal-level scaffolds. These chromosome-level scaffolds, confirmed by Hi-C data, are named according to size ( Figure 5; Table 3). During curation, the X chromosome could not be identified, due to lack of a comparator.

**Table 3.** Chromosomal pseudomolecules in the genome assembly of *Lithobius variegatus*, qcLitVari1.

INSDC accession	Name	Length (Mb)	GC%
OZ222603.1	1	271.1	40
OZ222604.1	2	144.17	40.5
OZ222605.1	3	92.49	40
OZ222606.1	4	86.49	40
OZ222607.1	5	84.09	40
OZ222608.1	6	82.29	39.5
OZ222609.1	7	78.49	40
OZ222610.1	8	78.18	40
OZ222611.1	9	75.15	40
OZ222612.1	10	74.65	40
OZ222613.1	11	73.91	40
OZ222614.1	12	66.28	40
OZ222615.1	13	61.29	40
OZ222616.1	14	55.76	40
OZ222617.1	15	52.07	40
OZ222618.1	16	51.79	40
OZ222619.1	17	48.57	40.5
OZ222620.1	18	47.91	40
OZ222621.1	19	47.37	40
OZ222622.1	20	42.99	40
OZ222623.1	21	40.12	40
OZ222624.1	22	36.78	40.5
OZ222625.1	23	33.6	40
OZ222626.1	MT	0.02	28

The mitochondrial genome was also assembled. This sequence is included as a contig in the multifasta file of the genome submission and as a standalone record.

### Assembly quality metrics

The estimated Quality Value (QV) and *k*-mer completeness metrics, along with BUSCO completeness scores, were calculated for each haplotype and the combined assembly. The QV reflects the base-level accuracy of the assembly, while *k*-mer completeness indicates the proportion of expected *k*-mers identified in the assembly. BUSCO scores provide a measure of completeness based on benchmarking universal single-copy orthologues.

For haplotype 1, the estimated QV is 62.4, and for haplotype 2, 62.2. When the two haplotypes are combined, the assembly achieves an estimated QV of 62.3. The *k*-mer recovery for haplotype 1 is 77.94%, and for haplotype 2 77.82%, while the combined haplotypes have a *k*-mer recovery of 99.30%. BUSCO v.5.5.0 analysis using the arthropoda_odb10 reference set ( *n* = 1,013) identified 97.7% of the expected gene set (single = 96.4%, duplicated = 1.3%) for haplotype 1.

Table 2 provides assembly metric benchmarks adapted from Rhie *et al.* (2021) and the Earth BioGenome Project Report on Assembly Standards September 2024. The haplotype 1 assembly achieves the EBP reference standard of **6.C.Q62.**

## Methods

### Sample acquisition and DNA barcoding

An adult *Lithobius variegatus* (specimen ID Ox001000, ToLID qcLitVari1) was collected from Wytham Woods, Oxfordshire, UK (latitude 51.772, longitude –1.338) on 2020-12-08 by potting. The specimen was collected by Liam Crowley (University of Oxford) and identified by Mark Telfer (University of Oxford) and preserved on dry ice.

The specimen used for RNA sequencing (specimen ID NHMUK014446397, ToLID qcLitVari2) was an adult female collected from Hever Castle, Kent, UK (latitude 51.188, longitude 0.12) on 2020-08-27 by handpicking. The specimen was collected by Lauren Hughes and Gregory Edgecombe (Natural History Museum), identified by Gregory Edgecombe and dry frozen.

The initial identification was verified by an additional DNA barcoding process according to the framework developed by Twyford *et al.* (2024). A small sample was dissected from the specimen and stored in ethanol, while the remaining parts were shipped on dry ice to the Wellcome Sanger Institute (WSI) ( Pereira *et al.*, 2022). The tissue was lysed, the COI marker region was amplified by PCR, and amplicons were sequenced and compared to the BOLD database, confirming the species identification ( Crowley *et al.*, 2023). Following whole genome sequence generation, the relevant DNA barcode region was also used alongside the initial barcoding data for sample tracking at the WSI ( Twyford *et al.*, 2024). The standard operating procedures for Darwin Tree of Life barcoding have been deposited on protocols.io ( Beasley *et al.*, 2023).

Metadata collection for samples adhered to the Darwin Tree of Life project standards described by Lawniczak *et al*. (2022).

### Nucleic acid extraction

The workflow for high molecular weight (HMW) DNA extraction at the Wellcome Sanger Institute (WSI) Tree of Life Core Laboratory includes a sequence of procedures: sample preparation and homogenisation, DNA extraction, fragmentation and purification. Detailed protocols are available on protocols.io ( Denton *et al.*, 2023b). The qcLitVari1 sample was prepared for DNA extraction by weighing and dissecting it on dry ice ( Jay *et al.*, 2023). Tissue from the anterior body was homogenised using a PowerMasher II tissue disruptor ( Denton *et al.*, 2023a). HMW DNA was extracted using the Automated MagAttract v1 protocol ( Sheerin *et al.*, 2023). For ultra-low input (ULI) PacBio sequencing, DNA was fragmented using the Covaris g-TUBE method ( Oatley *et al.*, 2023). Sheared DNA was purified by solid-phase reversible immobilisation, using AMPure PB beads to eliminate shorter fragments and concentrate the DNA ( Strickland *et al.*, 2023). The concentration of the sheared and purified DNA was assessed using a Nanodrop spectrophotometer and a Qubit Fluorometer using the Qubit dsDNA High Sensitivity Assay kit. The fragment size distribution was evaluated by running the sample on the FemtoPulse system.

RNA was extracted from mid-body tissue of qcLitVari2 in the Tree of Life Laboratory at the WSI using the RNA Extraction: Automated MagMax™ *mir*Vana protocol ( do Amaral *et al.*, 2023). The RNA concentration was assessed using a Nanodrop spectrophotometer and a Qubit Fluorometer using the Qubit RNA Broad-Range Assay kit. Analysis of the integrity of the RNA was done using the Agilent RNA 6000 Pico Kit and Eukaryotic Total RNA assay.

### Hi-C sample preparation and crosslinking

Hi-C data were generated from the head of the qcLitVari1 sample, using the Arima-HiC v2 kit (Arima Genomics) with 20–50mg of frozen tissue (stored at –80 °C). As per manufacturer’s instructions, tissue was fixed, and the DNA crosslinked using a TC buffer with 22% formaldehyde concentration, and a final formaldehyde concentration of 2%. The tissue was then homogenised using the Power Masher-II (Diagnocine). The crosslinked DNA was digested using a restriction enzyme master mix, then biotinylated and ligated. A clean up was performed with SPRIselect beads prior to library preparation. DNA concentration was quantified using the Qubit Fluorometer v4.0 (Thermo Fisher Scientific) and Qubit HS Assay Kit and sample biotinylation percentage was estimated using the Arima-HiC v2 QC beads.

### Library preparation and sequencing

Library preparation and sequencing were performed at the WSI Scientific Operations core.

***PacBio HiFi***

A ULI library was prepared using PacBio SMRTbell® Express Template Prep Kit 2.0 and PacBio SMRTbell® gDNA Sample Amplification Kit. To begin, samples were normalised to 20 ng of DNA. Initial removal of single-strand overhangs, DNA damage repair, and end repair/A-tailing were performed per manufacturer’s instructions. From the SMRTbell® gDNA Sample Amplification Kit, amplification adapters were then ligated. A 0.85X pre-PCR clean-up was performed with Promega ProNex beads and the sample was then divided into two for a dual PCR. PCR reactions A and B each followed the PCR programs as described in the manufacturer’s protocol. A 0.85X post-PCR clean-up was performed with ProNex beads for PCR reactions A and B and DNA concentration was quantified using the Qubit Fluorometer v4.0 (Thermo Fisher Scientific) and Qubit HS Assay Kit and fragment size analysis was carried out using the Agilent Femto Pulse Automated Pulsed Field CE Instrument (Agilent Technologies) and gDNA 55kb BAC analysis kit. PCR reactions A and B were then pooled, ensuring the total mass was ≥500 ng in 47.4 μl. The pooled sample then repeated the process for DNA damage repair, end repair/A-tailing and additional hairpin adapter ligation. A 1X clean-up was performed with ProNex beads and DNA concentration was quantified using the Qubit and fragment size analysis was carried out using the Agilent Femto Pulse Automated Pulsed Field CE Instrument (Agilent Technologies). Size selection was performed using the PippinHT system (Sage Science) with target fragment size determined by analysis from the Femto Pulse, usually a value between 4000 and 9000 bp. Size-selected libraries were then cleaned-up using1.0X ProNex beads and normalised to 2 nM before proceeding to sequencing.

Samples were sequenced using the Sequel IIe system (Pacific Biosciences, California, USA). The concentration of the library loaded onto the Sequel IIe was in the range 40–135 pM. The SMRT link software, a PacBio web-based end-to-end workflow manager, was used to set-up and monitor the run, as well as perform primary and secondary analysis of the data upon completion.

***Hi-C library preparation, amplification and sequencing***

Biotinylated DNA constructs were fragmented using the Covaris E220 sonicator (Covaris) and size-selected using SPRISelect beads to 400–600 bp. The DNA was enriched using the Arima-HiC v2 kit Enrichment beads. The NEBNext Ultra II DNA Library Prep Kit (New England Biolabs) was used for end repair, dA-tailing, and adapter ligation. This follows a modified NEBNext Ultra II DNA Library Prep protocol where library preparation occurs while DNA is bound to the Enrichment beads. Library PCR amplification was carried out using KAPA HiFi HotStart mix and a custom IDT UDI (Unique Dual Index) 96 barcode plate (Integrated DNA Technologies). Depending on sample concentration and biotinylation percentage determined at the crosslinking stage, samples were run for 10–16 PCR cycles. Post-PCR, samples were cleaned up using SPRISelect beads. The libraries were quantified using the Accuclear Ultra High Sensitivity dsDNA Standards Assay kit (Biotium) and normalised to 10 ng/μL before sequencing. Hi-C sequencing was performed an Illumina NovaSeq 6000 instrument using paired-end sequencing with a read length of 150 bp.

***RNA***

Poly(A) RNA-Seq libraries were constructed using the NEB Ultra II RNA Library Prep kit, following the manufacturer’s instructions. RNA sequencing was performed on the Illumina HiSeq 4000 instrument.

### Genome assembly, curation and evaluation

***Assembly***

Prior to assembly of the PacBio HiFi reads, a database of *k*-mer counts ( *k* = 31) was generated from the filtered reads using FastK. GenomeScope2 ( Ranallo-Benavidez *et al.*, 2020) was used to analyse the *k*-mer frequency distributions, providing estimates of genome size, heterozygosity, and repeat content.

The HiFi reads were assembled using Hifiasm in Hi-C phasing mode ( Cheng *et al.*, 2021; Cheng *et al.*, 2022), resulting in a pair of haplotype-resolved assemblies. The Hi-C reads ( Rao *et al.*, 2014) were mapped to the primary contigs using bwa-mem2 ( Vasimuddin *et al.*, 2019). The contigs were further scaffolded using the provided Hi-C datain YaHS ( Zhou *et al.*, 2023) using the --break option for handling potential misassemblies. The scaffolded assemblies were evaluated using Gfastats ( Formenti *et al.*, 2022), BUSCO ( Manni *et al.*, 2021) and MERQURY.FK ( Rhie *et al.*, 2020).

The mitochondrial genome was assembled using MitoHiFi ( Uliano-Silva *et al.*, 2023), which runs MitoFinder ( Allio *et al.*, 2020) and uses these annotations to select the final mitochondrial contig and to ensure the general quality of the sequence.

***Assembly curation***

The assembly was decontaminated using the Assembly Screen for Cobionts and Contaminants (ASCC) pipeline. Flat files and maps used in curation were generated via the TreeVal pipeline ( Pointon *et al.*, 2023). Manual curation was conducted primarily in PretextView ( Harry, 2022) and HiGlass ( Kerpedjiev *et al.*, 2018), with additional insights provided by JBrowse2 ( Diesh *et al.*, 2023). Scaffolds were visually inspected and corrected as described by Howe *et al*. (2021). Any identified contamination, missed joins, and mis-joins were amended, and duplicate sequences were tagged and removed. The curation process is documented at https://gitlab.com/wtsi-grit/rapid-curation.

***Assembly quality assessment***

The Merqury.FK tool ( Rhie *et al.*, 2020), run in a Singularity container ( Kurtzer *et al.*, 2017), was used to evaluate *k*-mer completeness and assembly quality for both haplotypes using the *k*-mer databases ( *k* = 31) computed prior to genome assembly. The analysis outputs included assembly QV scores and completeness statistics.

The genome was analysed using the BlobToolKit pipeline, a Nextflow ( Di Tommaso *et al.*, 2017) implementation of the earlier Snakemake BlobToolKit pipeline ( Challis *et al.*, 2020). The pipeline aligns PacBio reads using minimap2 ( Li, 2018) and SAMtools ( Danecek *et al.*, 2021) to generate coverage tracks. Simultaneously, it queries the GoaT database ( Challis *et al.*, 2023) to identify relevant BUSCO lineages and runs BUSCO ( Manni *et al.*, 2021). For the three domain-level BUSCO lineages, BUSCO genes are aligned to the UniProt Reference Proteomes database ( Bateman *et al.*, 2023) using DIAMOND blastp ( Buchfink *et al.*, 2021). The genome is divided into chunks based on the density of BUSCO genes from the closest taxonomic lineage, and each chunk is aligned to the UniProt Reference Proteomes database with DIAMOND blastx. Sequences without hits are chunked using seqtk and aligned to the NT database with blastn ( Altschul *et al.*, 1990). The BlobToolKit suite consolidates all outputs into a blobdir for visualisation.

The BlobToolKit pipeline was developed using nf-core tooling ( Ewels *et al.*, 2020) and MultiQC ( Ewels *et al.*, 2016), with package management via Conda and Bioconda ( Grüning *et al.*, 2018), and containerisation through Docker ( Merkel, 2014) and Singularity ( Kurtzer *et al.*, 2017).

Table 4 contains a list of relevant software tool versions and sources. The Tree of Life pipelines can be accessed via this page: https://pipelines.tol.sanger.ac.uk/pipelines.

**Table 4.** Software tools: versions and sources.

Software tool	Version	Source
BLAST	2.14.0	ftp://ftp.ncbi.nlm.nih.gov/blast/executables/blast+/
BlobToolKit	4.3.9	https://github.com/blobtoolkit/blobtoolkit
BUSCO	5.5.0	https://gitlab.com/ezlab/busco
bwa-mem2	2.2.1	https://github.com/bwa-mem2/bwa-mem2
DIAMOND	2.1.8	https://github.com/bbuchfink/diamond
fasta_windows	0.2.4	https://github.com/tolkit/fasta_windows
FastK	666652151335353eef2fcd58880bcef5bc2928e1	https://github.com/thegenemyers/FASTK
Gfastats	1.3.6	https://github.com/vgl-hub/gfastats
GoaT CLI	0.2.5	https://github.com/genomehubs/goat-cli
Hifiasm	0.19.8-r603	https://github.com/chhylp123/hifiasm
HiGlass	44086069ee7d4d3f6f3f0012569789ec138f42b84 aa44357826c0b6753eb28de	https://github.com/higlass/higlass
MerquryFK	d00d98157618f4e8d1a9190026b19b471055b2 2e	https://github.com/thegenemyers/MERQURY.FK
Minimap2	2.24-r1122	https://github.com/lh3/minimap2
MitoHiFi	3	https://github.com/marcelauliano/MitoHiFi
MultiQC	1.14, 1.17, and 1.18	https://github.com/MultiQC/MultiQC
Nextflow	23.10.0	https://github.com/nextflow-io/nextflow
PretextView	0.2.5	https://github.com/sanger-tol/PretextView
samtools	1.19.2	https://github.com/samtools/samtools
sanger-tol/ascc	-	https://github.com/sanger-tol/ascc
sanger-tol/blobtoolkit	0.6.0	https://github.com/sanger-tol/blobtoolkit
Seqtk	1.3	https://github.com/lh3/seqtk
Singularity	3.9.0	https://github.com/sylabs/singularity
TreeVal	1.2.0	https://github.com/sanger-tol/treeval
YaHS	1.2a.2	https://github.com/c-zhou/yahs

### Wellcome Sanger Institute – Legal and Governance

The materials that have contributed to this genome note have been supplied by a Darwin Tree of Life Partner. The submission of materials by a Darwin Tree of Life Partner is subject to the **‘Darwin Tree of Life Project Sampling Code of Practice’**, which can be found in full on the Darwin Tree of Life website here. By agreeing with and signing up to the Sampling Code of Practice, the Darwin Tree of Life Partner agrees they will meet the legal and ethical requirements and standards set out within this document in respect of all samples acquired for, and supplied to, the Darwin Tree of Life Project.

Further, the Wellcome Sanger Institute employs a process whereby due diligence is carried out proportionate to the nature of the materials themselves, and the circumstances under which they have been/are to be collected and provided for use. The purpose of this is to address and mitigate any potential legal and/or ethical implications of receipt and use of the materials as part of the research project, and to ensure that in doing so we align with best practice wherever possible. The overarching areas of consideration are:

•   Ethical review of provenance and sourcing of the material

•   Legality of collection, transfer and use (national and international) 

Each transfer of samples is further undertaken according to a Research Collaboration Agreement or Material Transfer Agreement entered into by the Darwin Tree of Life Partner, Genome Research Limited (operating as the Wellcome Sanger Institute), and in some circumstances other Darwin Tree of Life collaborators.

## Data Availability

European Nucleotide Archive: Lithobius variegatus (banded centipede). Accession number PRJEB77497; https://identifiers.org/ena.embl/PRJEB77497. The genome sequence is released openly for reuse. The *Lithobius variegatus* genome sequencing initiative is part of the Darwin Tree of Life Project (PRJEB40665). All raw sequence data and the assembly have been deposited in INSDC databases. The genome will be annotated using available RNA-Seq data and presented through the Ensembl pipeline at the European Bioinformatics Institute. Raw data and assembly accession identifiers are reported in Table 1 and Table 2.

## Author information

Members of the University of Oxford and Wytham Woods Genome Acquisition Lab are listed here: https://doi.org/10.5281/zenodo.12157525.

Members of the Natural History Museum Genome Acquisition Lab are listed here: https://doi.org/10.5281/zenodo.12159242.

Members of the Darwin Tree of Life Barcoding collective are listed here: https://doi.org/10.5281/zenodo.12158331.

Members of the Wellcome Sanger Institute Tree of Life Management, Samples and Laboratory team are listed here: https://doi.org/10.5281/zenodo.12162482.

Members of Wellcome Sanger Institute Scientific Operations: Sequencing Operations are listed here: https://doi.org/10.5281/zenodo.12165051.

Members of the Wellcome Sanger Institute Tree of Life Core Informatics team are listed here: https://doi.org/10.5281/zenodo.12160324.

Members of the Tree of Life Core Informatics collective are listed here: https://doi.org/10.5281/zenodo.12205391.

Members of the Darwin Tree of Life Consortium are listed here: https://doi.org/10.5281/zenodo.4783558.

## References

[ref-1] Allio R Schomaker-Bastos A Romiguier J : MitoFinder: efficient automated large-scale extraction of mitogenomic data in target enrichment phylogenomics. *Mol Ecol Resour.* 2020;20(4):892–905. 10.1111/1755-0998.13160 32243090 PMC7497042

[ref-2] Altschul SF Gish W Miller W : Basic Local Alignment Search Tool. *J Mol Biol.* 1990;215(3):403–410. 10.1016/S0022-2836(05)80360-2 2231712

[ref-3] Barber AD : Key to the identification of British centipedes.Field Studies Council,2008. Reference Source

[ref-4] Barber AD : Atlas of the centipedes of Britain and Ireland.Field Studies Council,2022.

[ref-5] Bateman A Martin MJ Orchard S : UniProt: the Universal Protein Knowledgebase in 2023. *Nucleic Acids Res.* 2023;51(D1):D523–D531. 10.1093/nar/gkac1052 36408920 PMC9825514

[ref-6] Beasley J Uhl R Forrest LL : DNA barcoding SOPs for the Darwin Tree of Life project. *protocols.io.* 2023; [Accessed 25 June 2024]. 10.17504/protocols.io.261ged91jv47/v1

[ref-7] Buchfink B Reuter K Drost HG : Sensitive protein alignments at Tree-of-Life scale using DIAMOND. *Nat Methods.* 2021;18(4):366–368. 10.1038/s41592-021-01101-x 33828273 PMC8026399

[ref-8] Challis R Kumar S Sotero-Caio C : Genomes on a Tree (GoaT): a versatile, scalable search engine for genomic and sequencing project metadata across the eukaryotic Tree of Life [version 1; peer review: 2 approved]. *Wellcome Open Res.* 2023;8:24. 10.12688/wellcomeopenres.18658.1 36864925 PMC9971660

[ref-9] Challis R Richards E Rajan J : BlobToolKit – interactive quality assessment of genome assemblies. *G3 (Bethesda).* 2020;10(4):1361–1374. 10.1534/g3.119.400908 32071071 PMC7144090

[ref-10] Cheng H Concepcion GT Feng X : Haplotype-resolved *de novo* assembly using phased assembly graphs with hifiasm. *Nat Methods.* 2021;18(2):170–175. 10.1038/s41592-020-01056-5 33526886 PMC7961889

[ref-11] Cheng H Jarvis ED Fedrigo O : Haplotype-resolved assembly of diploid genomes without parental data. *Nat Biotechnol.* 2022;40(9):1332–1335. 10.1038/s41587-022-01261-x 35332338 PMC9464699

[ref-12] Chipman AD Ferrier DEK Brena C : The first myriapod genome sequence reveals conservative arthropod gene content and genome organisation in the centipede *Strigamia maritima.* *PLoS Biol.* 2014;12(11): e1002005. 10.1371/journal.pbio.1002005 25423365 PMC4244043

[ref-13] Collins G Schneider C Boštjančić LL : MetaInvert: a new soil invertebrate genome resource provides insights into below-ground biodiversity and evolution. *Res Sq.* 2023; [Accessed 17 August 2023]. 10.21203/rs.3.rs-2706746/v1 PMC10709333 38066075

[ref-14] Crowley L Allen H Barnes I : A sampling strategy for genome sequencing the British terrestrial arthropod fauna [version 1; peer review: 2 approved]. *Wellcome Open Res.* 2023;8:123. 10.12688/wellcomeopenres.18925.1 37408610 PMC10318377

[ref-15] Danecek P Bonfield JK Liddle J : Twelve years of SAMtools and BCFtools. *GigaScience.* 2021;10(2): giab008. 10.1093/gigascience/giab008 33590861 PMC7931819

[ref-16] Denton A Oatley G Cornwell C : Sanger Tree of Life sample homogenisation: PowerMash. *protocols.io.* 2023a. 10.17504/protocols.io.5qpvo3r19v4o/v1

[ref-17] Denton A Yatsenko H Jay J : Sanger Tree of Life wet laboratory protocol collection V.1. *protocols.io.* 2023b. 10.17504/protocols.io.8epv5xxy6g1b/v1

[ref-18] Di Tommaso P Chatzou M Floden EW : Nextflow enables reproducible computational workflows. *Nat Biotechnol.* 2017;35(4):316–319. 10.1038/nbt.3820 28398311

[ref-19] Diesh C Stevens GJ Xie P : JBrowse 2: a modular genome browser with views of synteny and structural variation. *Genome Biol.* 2023;24(1): 74. 10.1186/s13059-023-02914-z 37069644 PMC10108523

[ref-20] do Amaral RJV Bates A Denton A : Sanger Tree of Life RNA extraction: automated MagMax ^TM^ mirVana. *protocols.io.* 2023. 10.17504/protocols.io.6qpvr36n3vmk/v1

[ref-21] Eason EH : Centipedes of the British Isles.Warne,1964. Reference Source

[ref-22] Eason EH Serra A : On the geographical distribution of *Lithobius variegatus* Leach, 184, and the identity of *Lithobius rubriceps* Newport, 1845 (Chilopoda: Lithobiomorpha). *J Nat Hist.* 1986;20:23–29.

[ref-23] Edgecombe GD Sivell D , Natural History Museum Genome Acquisition Lab, : The genome sequence of the centipede *Strigamia acuminata* (Leach, 1816) [version 1; peer review: 3 approved]. *Wellcome Open Res.* 2023;8:420. 10.12688/wellcomeopenres.19941.1 37808388 PMC10556566

[ref-25] Ewels P Magnusson M Lundin S : MultiQC: summarize analysis results for multiple tools and samples in a single report. *Bioinformatics.* 2016;32(19):3047–3048. 10.1093/bioinformatics/btw354 27312411 PMC5039924

[ref-24] Ewels PA Peltzer A Fillinger S : The nf-core framework for community-curated bioinformatics pipelines. *Nat Biotechnol.* 2020;38(3):276–278. 10.1038/s41587-020-0439-x 32055031

[ref-26] Formenti G Abueg L Brajuka A : Gfastats: conversion, evaluation and manipulation of genome sequences using assembly graphs. *Bioinformatics.* 2022;38(17):4214–4216. 10.1093/bioinformatics/btac460 35799367 PMC9438950

[ref-27] Grüning B Dale R Sjödin A : Bioconda: sustainable and comprehensive software distribution for the life sciences. *Nat Methods.* 2018;15(7):475–476. 10.1038/s41592-018-0046-7 29967506 PMC11070151

[ref-28] Harry E : PretextView (Paired REad TEXTure Viewer): a desktop application for viewing pretext contact maps.2022. Reference Source

[ref-29] Howe K Chow W Collins J : Significantly improving the quality of genome assemblies through curation. *GigaScience.* 2021;10(1): giaa153. 10.1093/gigascience/giaa153 33420778 PMC7794651

[ref-30] Iorio E : Catalogue biogéographique et taxonomique des chilopodes (Chilopoda) de la France métropolitaine. *Memoires de la Société linnéene de Bordeaux.* 2014;15. Reference Source

[ref-31] Jay J Yatsenko H Narváez-Gómez JP : Sanger Tree of Life sample preparation: triage and dissection. *protocols.io.* 2023. 10.17504/protocols.io.x54v9prmqg3e/v1

[ref-32] Kerpedjiev P Abdennur N Lekschas F : HiGlass: web-based visual exploration and analysis of genome interaction maps. *Genome Biol.* 2018;19(1): 125. 10.1186/s13059-018-1486-1 30143029 PMC6109259

[ref-33] Kurtzer GM Sochat V Bauer MW : Singularity: scientific containers for mobility of compute. *PLoS One.* 2017;12(5): e0177459. 10.1371/journal.pone.0177459 28494014 PMC5426675

[ref-34] Lawniczak MKN Davey RP Rajan J : Specimen and sample metadata standards for biodiversity genomics: a proposal from the Darwin Tree of Life project [version 1; peer review: 2 approved with reservations]. *Wellcome Open Res.* 2022;7:187. 10.12688/wellcomeopenres.17605.1

[ref-35] Leach WE : Crustaceology.In: Brewster, D. (ed.) *The Edinburgh encyclopaedia.*Edinburgh: Balfour,1813;7:385–437,765–766.

[ref-36] Li H : Minimap2: pairwise alignment for nucleotide sequences. *Bioinformatics.* 2018;34(18):3094–3100. 10.1093/bioinformatics/bty191 29750242 PMC6137996

[ref-37] Manni M Berkeley MR Seppey M : BUSCO update: novel and streamlined workflows along with broader and deeper phylogenetic coverage for scoring of eukaryotic, prokaryotic, and viral genomes. *Mol Biol Evol.* 2021;38(10):4647–4654. 10.1093/molbev/msab199 34320186 PMC8476166

[ref-38] Merkel D : Docker: lightweight Linux containers for consistent development and deployment. *Linux J.* 2014;2014(239): 2. [Accessed 2 April 2024]. Reference Source

[ref-39] Oatley G Sampaio F Kitchin L : Sanger Tree of Life HMW DNA fragmentation: covaris g-TUBE for ULI PacBio. *protocols.io.* 2023; [Accessed 13 June 2024]. 10.17504/protocols.io.q26g7pm81gwz/v1

[ref-40] Pereira L Sivell O Sivess L : DToL Taxon-specific Standard Operating Procedure for the terrestrial and freshwater arthropods working group.2022. 10.17504/protocols.io.261gennyog47/v1

[ref-41] Pointon DL Eagles W Sims Y : sanger-tol/treeval v1.0.0 – Ancient Atlantis. 2023. 10.5281/zenodo.10047654

[ref-42] Ranallo-Benavidez TR Jaron KS Schatz MC : GenomeScope 2.0 and Smudgeplot for reference-free profiling of polyploid genomes. *Nat Commun.* 2020;11(1): 1432. 10.1038/s41467-020-14998-3 32188846 PMC7080791

[ref-43] Rao SSP Huntley MH Durand NC : A 3D map of the human genome at kilobase resolution reveals principles of chromatin looping. *Cell.* 2014;159(7):1665–1680. 10.1016/j.cell.2014.11.021 25497547 PMC5635824

[ref-44] Rhie A McCarthy SA Fedrigo O : Towards complete and error-free genome assemblies of all vertebrate species. *Nature.* 2021;592(7856):737–746. 10.1038/s41586-021-03451-0 33911273 PMC8081667

[ref-45] Rhie A Walenz BP Koren S : Merqury: reference-free quality, completeness, and phasing assessment for genome assemblies. *Genome Biol.* 2020;21(1): 245. 10.1186/s13059-020-02134-9 32928274 PMC7488777

[ref-46] Roberts H : An ecological study of the arthropods of a mixed beech-oak woodland, with particular reference to Lithobiidae.University of Southampton,1956. Reference Source

[ref-47] Sheerin E Sampaio F Oatley G : Sanger Tree of Life HMW DNA extraction: automated MagAttract v.1. *protocols.io.* 2023. 10.17504/protocols.io.x54v9p2z1g3e/v1

[ref-48] So WL Nong W Xie Y : Myriapod genomes reveal ancestral horizontal gene transfer and hormonal gene loss in millipedes. *Nat Commun.* 2022;13(1): 3010. 10.1038/s41467-022-30690-0 35637228 PMC9151784

[ref-49] Strickland M Cornwell C Howard C : Sanger Tree of Life fragmented DNA clean up: manual SPRI. *protocols.io.* 2023. 10.17504/protocols.io.kxygx3y1dg8j/v1

[ref-50] Twyford AD Beasley J Barnes I : A DNA barcoding framework for taxonomic verification in the Darwin Tree of Life project [version 1; peer review: 2 approved]. *Wellcome Open Res.* 2024;9:339. 10.12688/wellcomeopenres.21143.1 39386966 PMC11462125

[ref-51] Uliano-Silva M Ferreira JGRN Krasheninnikova K : MitoHiFi: a python pipeline for mitochondrial genome assembly from PacBio high fidelity reads. *BMC Bioinformatics.* 2023;24(1): 288. 10.1186/s12859-023-05385-y 37464285 PMC10354987

[ref-52] Vasimuddin M Misra S Li H : Efficient architecture-aware acceleration of BWA-MEM for multicore systems.In: *2019 IEEE International Parallel and Distributed Processing Symposium (IPDPS).*IEEE,2019;314–324. 10.1109/IPDPS.2019.00041

[ref-53] Zhang L Zhang K Yang F : Chromosome-level genome of *Scolopendra mutilans* provides insights into its evolution. *Integr Zool.* 2024. 10.1111/1749-4877.12871 39075924

[ref-54] Zhou C McCarthy SA Durbin R : YaHS: yet another Hi-C scaffolding tool. *Bioinformatics.* 2023;39(1): btac808. 10.1093/bioinformatics/btac808 36525368 PMC9848053

