# Supplementary material for: The genome sequence of the banded centipede, Lithobius variegatus Leach, 1814
Source: Wellcome Open Res. 2025 Jun 2;10:285. [Version 1] doi: 10.12688/wellcomeopenres.24329.1 (PMC12368494; doi:10.12688/wellcomeopenres.24329.1)
Supplement: Peer review history

# Reviewer report: Reviewer response for version 1

**DOI:** 10.21956/wellcomeopenres.26830.r127605

**Published:** 2025-08-20

**Authors:** Ralf Janssen

**Affiliations:** 1 Uppsala University, Villavägen, Sweden

This Data Note reports on the genome sequencing of the centipede Lithobius variegatus. The quality and coverage of the sequenced genome (two haplotypes) are good and may provide the basis for further research on this species. Beyond that, these new high-quality data can be used for comparative genomic studies which is interesting for myriapod/arthropod researchers and (in a broader context) for evolutionary biologists alike.

 I understand that this paper is a Data Note that presents the methods and the pipeline(s) used to gain and analyse the data. 

 It would, however, be interesting and helpful to have a bit more graphical insights into the quality of the data. One could for example add some data on Hox gene clustering which would give some instant insights into the robustness of the chromosome assembly (assuming that there is a complete Hox cluster). Likewise one could add data on other clusters such as the NK gene cluster that has been in the focus of attention lately.

 It could also be highlighted if there are any unexpected gene losses or duplications.

 But maybe the authors plan to expand on their analysis in follow up papers instead.

 Apart from this, I believe that this is a very solid paper (as far as I can judge given my limited insights into genome sequencing and assembly techniques). 

 The BUSCO analysis appears to be a good proxy for the quality and coverage of the data. A result of 97,7% is very good (also when compared to other genome-sequencing approaches). 

 Therefore, I recommend this Data Note paper for indexing, either in its current form, or with some additional “biological” data such as Hox gene content and clustering.

Are sufficient details of methods and materials provided to allow replication by others?

Yes

Is the rationale for creating the dataset(s) clearly described?

Yes

Are the datasets clearly presented in a useable and accessible format?

Yes

Are the protocols appropriate and is the work technically sound?

Yes

Reviewer Expertise:

Developmental Biology, Evolutionary Biology, Arthropoda including Myriapoda, Onychophora, Gene expression analysis, Transcriptomics, Single-Cell-Sequencing, Minor insights into Genomics

I confirm that I have read this submission and believe that I have an appropriate level of expertise to confirm that it is of an acceptable scientific standard.

# Reviewer report: Reviewer response for version 1

**DOI:** 10.21956/wellcomeopenres.26830.r125017

**Published:** 2025-06-23

**Authors:** Wai Lok So

**Affiliations:** 1 The Chinese University of Hong Kong, Hong Kong, China

Edgecombe et al. produced a high quality genomic resource for the banded centipede, *Lithobius variegatus*. The genome was sequenced on the PacBio platform and further scaffolded with Hi-C data. The resultant assembled genome consists of 2 haplotype, one mainly contains 23 chromosomal pseudomolecules, and the other one was assembled to scaffold level. In addition, a 17.44kb mitochondrial genome was also sequenced and assembled. The sequencing and assembly methods used by the authors are standard and scientifically sound. A few points need to be addressed for further enhancement of the manuscript:

 1. Can the sex of the sample (qcLitVari1) used for PacBio sequencing and Hi-C be identified?

 2. Has the gut of the samples being removed before the nucleic acid extraction?

Are sufficient details of methods and materials provided to allow replication by others?

Yes

Is the rationale for creating the dataset(s) clearly described?

Yes

Are the datasets clearly presented in a useable and accessible format?

Yes

Are the protocols appropriate and is the work technically sound?

Yes

Reviewer Expertise:

myriapods, soil biodiversity, evolution, genome

I confirm that I have read this submission and believe that I have an appropriate level of expertise to confirm that it is of an acceptable scientific standard.
